# Trends and Social Differences in Alcohol Consumption during the Postcommunist Transition in Lithuania

**DOI:** 10.1100/2012/615183

**Published:** 2012-04-29

**Authors:** Jurate Klumbiene, Darius Kalasauskas, Janina Petkeviciene, Aurelijus Veryga, Edita Sakyte

**Affiliations:** Health Research Institute, Faculty of Public Health, Academy of Medicine, Lithuanian University of Health Sciences, 50009 Kaunas, Lithuania

## Abstract

The aim of the study was to evaluate the trends and social differences in consumption of various types of alcoholic beverages in Lithuania over the postcommunist transition period (1994–2010). The data were obtained from nine nationally representative postal surveys of Lithuanian population aged 20–64 conducted every second year (*n* = 17154). Prevalence of regular (at least once a week) consumption of beer, wine, or strong alcoholic beverages and the amount of alcohol consumed per week were examined. Regular beer drinking as well as the amounts consumed increased considerably in both genders. The increase in regular consumption of strong alcohol was found among women. Sociodemographic patterning of regular alcohol drinking was more evident in women than in men. In women, young age and high education were associated with frequent regular drinking of wine and beer. Social differences in regular alcohol drinking should be considered in further development of national alcohol control policy in Lithuania.

## 1. Introduction

Alcohol consumption is one of the main factors contributing to premature mortality and morbidity, which account for 3.8% deaths and 4.6% disability-adjusted life years worldwide [1]. Eastern European region has higher mortality and morbidity from noncommunicable diseases and large gender gap compared to Western European countries [2]. About 25% of difference in men, life expectancy between Western and Eastern European countries can be attributed to alcohol consumption [3]. 

Lithuania is characterized by having one of the most detrimental drinking patterns in European Union (EU) [4]. Alcohol-attributable years of potential life lost in Lithuania are considerably higher compared to Western European countries [5]. In 2010, alcohol consumption in Lithuania was 11.3 L of pure alcohol per capita [6]. During the period 1990–2010, per capita consumption of pure alcohol more than doubled.

A reduction of alcohol consumption was one of the aims of Lithuanian Health Programme approved by parliament in 1998 [7]. However, after regaining independence in 1990, liberalization of alcohol control policy by abolishment of state monopoly for production of strong beverages combined with alcohol industry privatization, reduction of alcohol excise tax, and extensive alcohol advertisement and sponsorship has lead to increased accessibility and consumption of alcohol. Recently (2008-2009), some evidence-based measures to reduce alcohol consumption were introduced: time limitation for off-premise sales of alcoholic beverages, restriction of alcohol advertisement on TV and radio, increase of excise tax, and so forth [8]. Little is known about how these measures influenced drinking patterns of Lithuanians. Because alcohol control measures were beverage specific, they might have different impact on consumption of various types of alcoholic beverages.

Relationship between social factors and alcohol consumption was found in many studies [9–12]. Most of them show that socially disadvantaged groups have heavier drinking patterns. Over the period of transition to market economy, a gap between social classes has widened in Lithuania [13]. This might have affected health behaviour, including alcohol drinking habits.

The aim of the study was to evaluate the trends and social differences in regular consumption of various types of alcoholic beverages in Lithuania during the period 1994−2010.

## 2. Methods

The data were obtained from nine cross-sectional postal surveys conducted within the framework of Finbalt Health Monitor project. The surveys in Lithuania have been carried out every second year since 1994. Each survey was based on a nationally representative random sample drawn from the national population register. The sample consisted of 3000 citizens aged 20–64 in 1994–2008 surveys and 4000 in the 2010 survey. The sampling unit was individual in all surveys and no measures were taken to substitute nonrespondents. The methodology and questionnaires were standardized [14]. The questionnaire, mailed between April and June with one reminder, has remained essentially unchanged over the years. Response rates were satisfactory in all surveys ranging from 54 to 74%. Total number of respondents was 17154 (7358 men and 9796 women). The characteristics of the study population are presented in Table 1. The Lithuanian Bioethics Committee approved all surveys.

The frequency of drinking beer, wine including sparkling wine, and strong alcohol was determined with the following questions: “How often do you consume strong alcohol, spirits?”, “How often do you consume wine or sparkling wine?”, and “How often do you consume beer?”. The possible responses were “every day,” “2-3 times a week,” “once a week,” “2-3 times a month,” “several times a year,” and “never.” Questions on frequency of drinking strong alcohol and beer were included in surveys since 1994. The questionnaire was supplemented by the question about frequency of wine or sparkling wine drinking in 1998. Regular consumption of each alcoholic beverage as well as any alcohol was considered as drinking at least once a week for both genders.

The volume of alcohol consumed per week was assessed with the question: “How many glasses (regular restaurant portions) or bottles of the following alcoholic beverages (beer, wine, or strong alcohol) have you drunk during the last week (7 days)?” The answers were used to calculate standard drinks for each type of alcoholic beverage and total amount of alcohol consumed per week. One standard drink was estimated to contain 12.7 mL or 10 g of pure alcohol. The respondents' 7-day volume of consumption of a given alcoholic beverage was determined by the following formula: cl (%/100)/1.27, where cl is the capacity of the given alcoholic beverage in centiliters and % is its alcohol content in percentages. The total volume of alcohol consumed in the previous seven days was calculated as a sum of standard drinks for beer, wine, and strong alcohol. Beer, wine or sparkling wine, and strong alcohol were assumed to contain 5%, 11%, and 40% of alcohol, respectively.

The sociodemographic determinants were gender, age, level of education, level of urbanization, and marital status. Education was measured by educational levels (primary, incomplete secondary, secondary, vocational school, college, and university). The respondents were categorized into three groups: persons with low (primary, incomplete secondary, secondary), medium (vocational school, college), or high (university) education level. According to the administrative classification of places of residence, the respondents were grouped as living in cities, towns, or villages. Marital status was dichotomized as “married or cohabiting” and “others” (single, divorced, or widowed).

Statistical analyses were performed using the statistical software package SPSS 19.0 for Windows. Data for men and women were analyzed separately. The data were weighted to match the age distribution of the Lithuanian population aged 20–64 in the year 2010. We calculated the proportions to estimate the prevalence of drinking each type of alcoholic beverages at least once a week. The association between time and frequency of alcohol consumption was not linear. Therefore, 95% confidence intervals were used to analyze the differences during the study period so that each time point could be compared to another. Normal approximation was used in calculation of 95% confidence intervals for standardized proportions.

Mean values, standard deviations, and medians of standard drinks from each type of alcoholic beverage and any alcohol consumed during the last week were calculated for respondents who had drunk at least one portion of any alcoholic beverage during the last seven days. The distribution of the analyzed variables did not meet criteria of normality checked by Kolmogorov-Smirnov test, therefore the differences between the groups were assessed applying Kruskal-Wallis analysis of variance.

Sociodemographic differences in regular consumption of beer, wine or sparkling wine, and strong alcohol were examined by applying logistic regression analysis. All models were applied separately for each type of alcoholic beverage among men and women. The odds of drinking a specific alcoholic beverage were calculated with adjustment for age, education, place of residence, and marital status. The overall effect was added first, followed by age, education, place of residence, and marital status. The results are presented as odds ratios (OR) and their confidence intervals (CI) in Tables 4 and 5.

## 3. Results

Regular drinking of strong alcoholic beverages was significantly more common in men, compared to women in all surveys (Tables 2 and 3). No major changes in the proportion of men consuming strong alcoholic beverages at least once a week were found during the period 1994–2008. The prevalence of regular drinking of this type of alcoholic beverage was significantly lower in 2010, compared to the years 1994 and 2000. The increase in women's regular drinking of strong alcohol was observed during the period 1994–2000; however, afterwards it remained stable. Wine or sparkling wine was less popular compared to other alcoholic beverages. The prevalence of regular wine drinking did not differ by sex during the whole study period. The proportion of regular wine or sparkling wine drinkers in men was higher in years 1998 and 2000, compared to other study periods (Table 2). During the whole study period, we did not find any systematic changes in the prevalence of regular wine or sparkling wine drinking among women (Table 3). According to our data, Lithuanian men for regular drinking chose beer from all alcoholic beverages most frequently. The proportion of regular beer drinkers among men increased significantly from 43.8% (95% CI: 40.3–47.4) in 1994 to 55.9% (95% CI: 52.8–59.0) in 2000 and remained stable in the following surveys. Similar trends were observed among women. In all surveys, the proportion of women who consumed beer regularly was 3–6-fold smaller compared to men. The trends of regular drinking prevalence of any kind of alcoholic beverage are presented in Tables 2 and 3. The data about all three alcoholic beverages (strong alcohol, beer, and wine or sparkling wine) were available only since 1998 when the supplementary question on wine was added to the questionnaire. In 1998, the proportion of men and women drinking any alcoholic beverage regularly was significantly lower than in the surveys conducted in 2000–2008.

Table 4 demonstrates age-adjusted mean and median of standard drinks consumed per week alcohol in men. Over the whole observation period, weekly consumption of strong alcohol and wine or sparkling wine has not changed in men. We found a statistically significant increase in amount of beer, converted to standard drinks, consumed from 8.0 (6.6) in 1994 to 10.4 (9.3) in 2010. Systematic but not significant increase in age-adjusted mean and median of standard drinks from total alcohol consumed by men was observed between 1994–1998 and 2006−2010. Since 1994, weekly wine or sparkling wine and beer consumption among women has increased significantly, while intake of strong alcohol has not changed (Table 5). In this group, the age-adjusted mean and median of standard drinks from total alcohol were two times higher in 2010 compared to 1994 and 1996.

**Figure 1:**
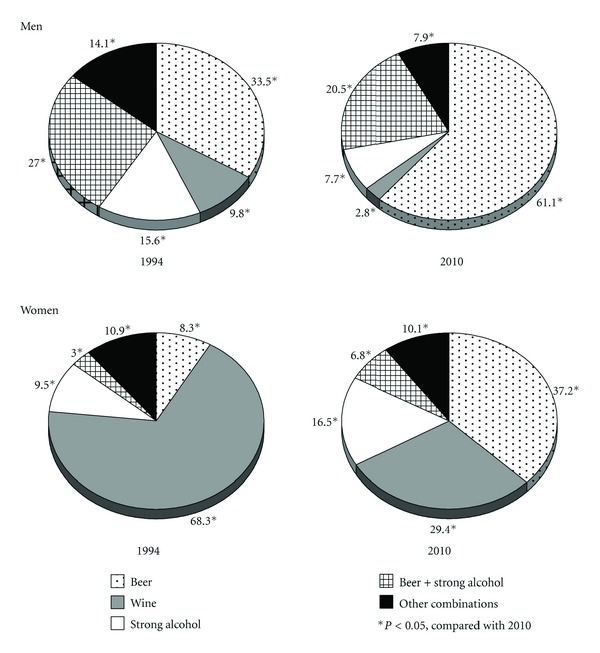
Proportion (%) of different types of alcoholic beverages consumed at least once a week single or in combination by gender in 1994 and 2010.

The frequency of specific alcoholic beverages or their combinations consumed by men and women during the last week in 1994 and 2010 is presented in Figure 1. In 1994, one-third of men (33.5%) and 8.3% of women reported consuming only beer. Over sixteen years, this proportion has increased approximately two times in men and four times in women (*P* < 0.05). The frequency of drinking exclusively wine or sparkling wine declined in both genders. However, the decrease was particularly evident in women (from 68.3% in 1994 to 29.4% in 2010). The proportion of men consuming only strong alcoholic beverages decreased, while such proportion in women increased during the observation period. Our results show that 27% of men in 1994 and 20.5% of men in 2010 used to drink beer and strong alcohol during the last week (*P* < 0.05). Such drinking pattern was not common among women.

Associations of regular alcohol consumption with age, education, place of residence, and marital status were explored in a model in which odds ratios within categories of each sociodemographic variable were fully adjusted for all variables (Tables 6 and 7). Among men, sociodemographic patterning of regular alcohol drinking differed depending on type of alcoholic beverage (Table 6). Regular consumption of strong alcoholic beverages was least common in the youngest age group, while regular beer drinking was least common in the oldest age group. Educational inequalities in regular alcohol drinking were found only for wine or sparkling wine: highly educated men consumed wine at least once a week more often compared to the less educated. Beer was more popular among married men and those living in cities.

Women demonstrated higher sociodemographic differences in regular alcohol drinking compared to men (Table 7). These inequalities were generally the same for each type of alcoholic beverage. Regular alcohol drinking was most common in the youngest age group and among women living in cities. Highly educated women used to drink strong alcohol and wine or sparkling wine more often than the less educated. Educational gradient was the steepest for wine drinking. There was no association between regular beer drinking and level of education among women.

## 4. Discussion

This study focused on the trends and sociodemographic differences in regular consumption of various types of alcoholic beverages in Lithuanian population aged 20–64. Our findings indicate that more than a half of men and approximately one-quarter of women consumed alcoholic beverages at least once a week. Beer became the most popular drink among men and women. Over sixteen years, beer drinking frequency and consumed amounts increased significantly in both sexes. In 2010, the number of standard drinks from beer and strong alcohol consumed per week was approximately the same.

The trends in regular alcohol consumption during the postcommunist transition varied depending on gender and type of alcoholic beverage used. The most significant increase in beer consumption occurred in the first decade of transition period (1990–2000). In Soviet period, the frequency of beer drinking was low probably due to limited variety of beers in the market and prevalent traditional vodka drinking culture. Liberalization of alcohol market in 1990s considerably increased the supply of different kinds of beer [6]. The great increase in availability of beer, forceful advertising, and affordable prices resulted in increased consumption of this alcoholic beverage. Similar trends in beer consumption have been reported in other Baltic countries [9, 10]. 

Over the study period, the prevalence of regular strong alcohol consumption and amount of wine consumed per week increased significantly among Lithuanian women but remained stable among men. In women, the number of standard drinks from total alcohol consumed per week has doubled since 1994. Gender gap in regular alcohol consumption decreased significantly during the study years. However, it still remained large in the last survey. Many studies across the world indicate that alcohol consumption among women is rising, especially among young women [10, 15–18]. This trend is very similar to expansion of tobacco epidemic; when men population is targeted at first and when the possibilities of expansion are exhausted, marketing strategy is reoriented to women, associating the product with emancipation, sexuality, and so forth [19].

Our study showed that younger men preferred beer, while regular use of strong alcohol was more popular in older age group. High prevalence of regular beer drinking among younger men could be explained by aggressive advertising and sophisticated marketing of beer, targeting mainly youth. In Lithuania, beer-producing companies are usually the main sponsors of various sport events, youth-oriented music concerts, and festivals. The promotional campaigns for beer as well as for other alcoholic beverages intensively use new technologies such as the internet, which is easily accessed by young people. Older generation follows old Soviet period tradition to drink spirits. The findings of an earlier study conducted by McKee et al. showed that weekly consumption of all alcoholic beverages decreased with age in Lithuanian adult population [20]. 

A majority of the studies on educational inequalities of alcohol use examined frequency of drinking any alcohol or heavy/binge drinking according to the educational level [9, 10, 21–23]. Our study focused on investigation of educational differences in regular drinking of various types of alcoholic beverages. The obtained data revealed consistent educational gradient in regular drinking of wine or sparkling wine in both genders: higher education level was associated with regular drinking of wine. During transition period, a wide range of new wines has come to Lithuanian market. Wine producers started aggressive advertising and marketing targeting people in high socioeconomic position. Wine became a fashionable drink and started to be perceived socially as a “modern” drinking habit. It should be noted that wine is relatively expensive compared to strong alcoholic beverages; therefore, it is more affordable for better educated people with higher income.

Several studies have reported inconsistent urban-rural differences in alcohol drinking in Baltic countries [10, 20, 24]. According to our data, urban women used to drink regularly all types of alcoholic beverages more often compared to women living in rural areas. This could be explained by modern urban lifestyle, higher exposition to alcohol advertisement, and higher income of urban women.

Several limitations of our study should be considered when interpreting the results. It is well known that survey respondents have a tendency to underreport their alcohol consumption [25–27]. However, it is likely that underreporting was the same in all surveys and could not affect the trends. The response rates of the surveys have been relatively high, but decreasing trend has, however, been observed over the study period. We did not have data on nonrespondents, but comparison of early and late respondents revealed only the slight difference between them [28]. However, it is likely that people with higher alcohol consumption are overrepresented among nonrespondents. Therefore, regular alcohol drinking might be more prevalent if nonrespondents had responded. Despite these drawbacks, this study has revealed several important findings.

Our study suggests that alcohol consumption in Lithuania has increased over postcommunist transition period, especially among women. Only slight decrease in the frequency of regular alcohol drinking was observed between 2008 and 2010. This decline may be a random variation, but it coincides with recent introduction of the measures to reduce alcohol consumption in Lithuania: restriction of alcohol advertisement, limitation on selling time, and increased excise tax [8]. In 2009, Lithuania experienced economic crisis, which was followed by significant decrease in purchase power of the population and might have an impact on alcohol consumption.

The evidence shows that policies aiming at limitation of the economic and physical availability of alcohol are effective in reducing alcohol-related harm. A meta-analysis of 112 studies proved an impact of price on alcohol consumption, with the effect greater in the longer than the shorter run [29]. A rise in alcohol prices leads to decrease in alcohol consumption and lowers alcohol-related harm, and vice versa. In Finland, after the reduction of alcohol prices in 2004, consumption of alcohol increased significantly especially in the population older than 45 years and among lower educated people [30]. Furthermore, restriction on days and hours of sale and increasing of minimum purchase age reduces alcohol-related harm [31, 32]. The studies have consistently reported correlations between increased exposure to advertising and greater likelihood of current drinking [33]. 

New evidence-based alcohol control measures such as total ban on alcohol advertisement, stronger restrictions on alcohol availability (selling of alcoholic beverages only in specialized shops), and raising of minimum purchase age for strong alcohol beverages till 21 year are going to be implemented in Lithuania in order to reduce alcohol consumption and alcohol-related harm.

## 5. Conclusions

Over postcommunist transition period, the remarkable increase in the frequency of regular beer drinking as well as the amounts consumed was observed in Lithuanian adult population. The prevalence of regular consumption of strong alcohol and the amount of wine consumed per week have increased significantly among women and remained stable among men. Sociodemographic patterning of regular alcohol drinking was more evident in women than in men and depended on the type of alcoholic beverage consumed. Young age and high education were associated with frequent regular drinking of wine and beer in women. Social differences in regular alcohol drinking should be considered in further development of national alcohol control policy in Lithuania.

## Figures and Tables

**Table 1:** Characteristics of study population (1994–2010).

Sociodemographic variables	Men (*n* = 7358)	Women (*n* = 9796)
Age		
20–34	32.5	31.0
35–49	37.4	36.7
50–64	30.2	32.3
Education		
High	18.3	25.4
Medium	34.4	36.6
Low	47.2	38.0
Place of residence		
Cities	42.4	46.0
Towns	27.3	28.7
Villages	30.3	25.3
Marital status		
Married	74.2	66.9
Others	25.8	33.1

**Table 2:** Age-adjusted prevalence and confidence intervals (CIs) of regular drinking of strong alcohol, wine^a^, and beer among men in 1994–2010.

Survey year	Strong alcohol		Wine		Beer		Any alcohol	
	%	95% CIs	%	95% CIs	%	95% CIs	%	95% CIs
1994	31.0	27.8–34.3	—	—	43.8	40.3–47.4	—	
1996	28.6	25.7–31.5	—	—	40.0	36.8–43.1	—	
1998	27.0	24.0–30.1	12.6	10.3–14.9	48.5	45.0–51.9	55.5	52.1–58.9
2000	33.8	30.8–36.8	13.4	11.2–15.5	55.9	52.8–59.0	62.6	59.6–65.6
2002	28.4	25.4–31.5	7.6	5.8–9.4	59.0	55.7–62.4	62.6	59.3–65.9
2004	27.9	24.7–31.1	6.9	5.0–8.7	52.5	48.9–56.0	58.2	54.7–61.6
2006	30.0	26.6–33.4	5.8	4.5–7.5	56.8	53.2–60.5	60.5	58.9–64.0
2008	28.6	25.3–31.9	9.9	7.7–12.1	53.2	49.6–56.9	59.8	56.3–63.4
2010	23.6	20.5–26.6	8.2	6.3–10.2	51.1	47.5–54.7	56.2	52.7–59.8

^a^Including sparkling wine.

**Table 3:** Age-adjusted prevalence and confidence intervals (CIs) of regular drinking of strong alcohol, wine^a^, and beer among women in 1994–2010.

Survey year	Strong alcohol		Wine		Beer		Any alcohol	
	%	95% CIs	%	95% CIs	%	95% CIs	%	95% CIs
1994	5.6	4.2–7.1	—	—	7.3	5.7–8.9	—	
1996	6.7	5.2–8.2	—	—	8.3	6.7–10.0	—	
1998	6.6	5.1–8.1	9.8	7.9–11.6	12.7	10.7–14.8	20.8	18.3–23.2
2000	11.5	9.7–13.3	11.5	9.7–13.3	18.3	16.1–20.5	25.9	23.4–28.4
2002	8.3	6.6–10.0	10.7	8.8–12.6	18.3	15.9–20.7	25.1	22.5–27.7
2004	10.2	8.4–12.1	7.7	6.0–9.3	16.8	14.5–19.1	24.0	21.4–26.6
2006	10.2	8.3–12.1	8.0	6.3–9.7	18.3	15.9–20.7	25.7	23.0–28.4
2008	12.4	10.3–14.4	14.1	11.9–16.3	14.5	12.3–16.6	28.4	25.6–31.1
2010	8.8	7.2–10.4	8.6	7.0–10.2	12.2	10.4–14.1	22.2	19.8–24.5

^a^Including sparkling wine.

**Table 4:** Mean, standard deviation (SD), and median of standard drinks^a^ consumed by men during the last week in 1994–2010.

Survey year	Strong alcohol		Wine		Beer		Total	
	Mean (SD)	Median	Mean (SD)	Median	Mean (SD)	Median	Mean (SD)	Median
1994	12.1 (9.7)	12.6	2.5 (2.9)	1.7	8.0 (6.6)	5.9	12.6 (12.8)	8.8
1996	11.9 (11.3)	12.6	2.7 (2.6)	1.7	8.1* (7.1)	5.9	11.8 (12.7)	7.9
1998	12.5 (10.6)	10.1	3.9 (3.7)	2.6	7.8* (6.6)	5.9	12.1 (12.3)	7.9
2000	12.5 (11.5)	10.1	5.8 (9.4)	2.6	8.9 (10.1)	5.9	13.7 (15.6)	9.8
2002	10.3 (9.1)	7.6	3.4 (5.4)	1.7	9.2 (8.0)	7.9	12.8 (12.1)	9.8
2004	12.7 (11.5)	8.8	5.9 (4.6)	3.5	8.8 (7.7)	7.9	12.9 (13.2)	7.9
2006	12.3 (10.2)	10.1	4.6 (4.6)	3.5	9.4 (8.6)	7.9	14.0 (13.5)	9.8
2008	12.2 (12.8)	7.6	2.2 (1.8)	1.7	10.1 (10.5)	7.9	14.1 (15.0)	9.8
2010	12.0 (12.1)	7.6	3.9 (4.5)	2.6	10.4 (9.3)	7.9	13.5 (13.0)	9.8

*P < 0.05 compared to 2010, Kruskal-Wallis test.

^a^Standard drinks are calculated separately for strong alcohol, wine or sparkling wine, beer, and total alcohol consumed per week.

**Table 5:** Mean, standard deviation (SD), and median of standard drinks^a^ consumed by women during the last week in 1994–2010.

Survey year	Strong alcohol		Wine		Beer		Total	
	Mean (SD)	Median	Mean (SD)	Median	Mean (SD)	Median	Mean (SD)	Median
1994	5.9 (5.3)	3.8	1.6* (1.3)	0.9	3.3* (1.8)	2.0	3.0 (4.1)*	1.7
1996	5.7 (4.6)	5.0	1.7* (1.6)	1.7	3.4* (2.4)	2.0	3.0 (3.6)*	1.7
1998	5.5 (3.8)	3.8	3.1 (4.9)	1.7	4.0* (3.8)	2.0	4.8 (5.2)	3.5
2000	5.4 (4.6)	5.0	2.3 (1.5)	1.7	3.7* (2.8)	2.0	4.7 (4.2)	3.7
2002	4.4 (3.8)	3.8	2.5 (2.0)	1.7	4.0* (3.3)	2.0	4.8 (4.4)	3.9
2004	5.2 (4.0)	3.8	2.6 (1.3)	2.6	4.6 (4.2)	3.9	5.4 (4.9)	3.9
2006	5.4 (4.8)	3.8	2.7 (1.6)	1.7	4.1 (3.2)	3.9	5.2 (4.7)	3.9
2008	3.9 (2.8)	2.5	2.8 (2.0)	2.6	4.9 (4.7)	3.9	5.0 (4.7)	3.8
2010	5.6 (4.3)	3.8	3.3 (3.0)	2.6	6.4 (6.5)	3.9	6.1 (6.0)	3.9

*P < 0.05 compared to 2010, Kruskal-Wallis test.

^a^Standard drinks are calculated separately for strong alcohol, wine or sparkling wine, beer, and total alcohol consumed per week.

**Table 6:** Regular drinking of strong alcohol, wine^a^, and beer among men by sociodemographic variables (odds ratios (ORs) and confidence intervals (CIs)).

Sociodemographic variables	Strong alcohol		Wine		Beer	
	ORs	95% CIs	ORs	95% CIs	ORs	95% CIs
Age						
20–34	1		1		1	
35–49	1.35	1.19–1.54	1.17	0.93–1.48	0.79	0.70–0.89
50–64	1.19	1.04–1.37	1.07	0.84–1.38	0.48	0.42–0.54
Education						
High	1		1		1	
Medium	1.09	0.94–1.27	0.59	0.47–0.74	0.95	0.83–1.08
Low	1.15	0.99–1.34	0.47	0.37–0.59	0.98	0.86–1.12
Place of residence						
Cities	1		1		1	
Towns	0.86	0.76–0.98	0.81	0.64–1.02	0.83	0.74–0.93
Villages	1.10	0.97–1.24	1.19	0.96–1.49	0.84	0.75–0.94
Marital status						
Married	1		1		1	
Others	1.06	0.94–1.20	1.06	0.85–1.33	0.81	0.72–0.91

^a^Including sparkling wine.

**Table 7:** Regular drinking of strong alcohol, wine^a^, and beer among women by sociodemographic variables (odds ratios (ORs) and confidence intervals (CIs)).

Sociodemographic variables	Strong alcohol		Wine		Beer	
	ORs	95% CIs	ORs	95% CIs	ORs	95% CIs
Age						
20–34	1		1		1	
35–49	0.91	0.77–1.07	0.83	0.70–0.98	0.70	0.61–0.80
50–64	0.62	0.51–0.75	0.34	0.27–0.44	0.35	0.30–0.42
Education						
High	1		1		1	
Medium	0.79	0.67–0.94	0.47	0.40–0.57	0.88	0.76–1.02
Low	0.68	0.56–0.82	0.25	0.20–0.31	0.88	0.75–1.03
Place of residence						
Cities	1		1		1	
Towns	0.76	0.64–0.91	0.66	0.55–0.80	0.79	0.69–0.92
Villages	0.79	0.66–0.95	0.62	0.49–0.77	0.81	0.70–0.95
Marital status						
Married	1		1		1	
Others	1.07	0.92–1.25	0.85	0.71–1.01	0.91	0.80–1.04

^a^Including sparkling wine.
